# Study of Variability of Waste Wood Samples Collected in a Panel Board Industry

**DOI:** 10.3389/fchem.2021.722090

**Published:** 2021-07-26

**Authors:** Manuela Mancini, Åsmund Rinnan

**Affiliations:** Department of Food Science, Faculty of Science, University of Copenhagen, Frederiksberg C, Denmark

**Keywords:** sampling, variability, NIR spectroscopy, nested analysis of variance, heterogeneity, PCA

## Abstract

Waste wood is becoming an appealing alternative material to virgin wood, and the main drivers are the increased demand for waste wood by the panel industry, the introduction of renewable energy policies, and the waste framework directive. In fact, the use of waste wood as a secondary resource is favored over both landfills and combustion. The best reuse and cascading use of the material are linked to its characteristics. That is why it is important to know the chemical composition and the variation in the properties of such a heterogeneous material. In this article, a sampling study was carried out in a panel board company located in the northern part of Italy. In order to investigate the heterogeneity of waste wood, all samples have been analyzed by near-infrared spectroscopy. Nested analysis of variance and principal component analysis have been used to evaluate the heterogeneity and the variation in sample properties. The approach gives information about how to ensure representative measurements and efficiently describe the variability of the material. The results suggest that it is important to have replicates or at least two subsamples for each lot and then measure each of these with at least 100 scans, in order to get representative measurements and describe the variability of the material. The determination of waste wood composition and variability is the focal point for improving the sorting process and increasing the reuse of waste wood, avoiding expensive landfills and risks for human health and the environment.

## Introduction

Wood is one of the oldest and highly exploited resources in several sectors (e.g., pulp, construction, and energy), but it is also a limited resource (Rettenmaier et al., 2008). Since the 1970s, wood consumption has increased continuously, and it is expected to do so in the future (FAO/ECE, 2012). At the end of the life cycle, wood utilization produces waste wood (WW). The term indicates wood or wood-containing post-consumer and post-use products from different sectors (packaging, furniture, construction and demolition, and industrial and commercial sectors) (Edo et al., 2016). A study has estimated that the European Union generates 50 million cubic meters of wood waste each year (Mantau, 2012), and nowadays, a large amount remains unused (Hakala, 2012).

The most relevant drivers of the growth of the waste wood trade are related to the increased demand for waste wood by the panel board industry (Mazzanti and Zoboli, 2013) (Bergeron, 2016). The European Union is promoting the reuse and recycling of the materials over the landfill (Waste Framework Directive, 2008/98/EC, European Parliament 2008) (Commission of the European Communities, 2008) and has introduced European renewable energy policies for mitigating greenhouse gas emissions (Röder and Thornley, 2018).

Because of the various sources of origin, WW composition presents high heterogeneity (Huron et al., 2017). In addition, it should be taken into account that its chemical composition, quality classes definition, and degree of contamination also change according to the countries and their different laws (Edo et al., 2016). Consequently, identifying the best-suited application and possible end-users is related to the assessment of the WW composition and quality characteristics.

Some studies have already examined the characterization of waste wood materials. Edo et al. have investigated the waste wood variability across time (Edo et al., 2016). They collected five hundred samples from an industrial heating plant during nine years and performed lab analysis to assess the material heterogeneity. The concentrations of the examined contaminants varied according to the sampling method, demonstrating the variability of the material. In another study, Moreno and Font have carried out a complete characterization of furniture waste wood and studied the differences in thermochemical conversion by performing pyrolysis tests (Moreno and Font, 2015). Huron et al. have performed an extensive characterization of various treated waste wood to evaluate their heterogeneity and assessment of suitability with combustion processes. Different samples were collected, including waste wood mixtures, specific waste wood classes, and untreated wood for comparison. Some parameters, such as heating value and composition in C, H, and O, did not vary significantly compared to those of untreated wood, while minor elements showed differences in relation to the chemical treatments of waste wood (Huron et al., 2017). Faraca et al. have investigated the quality of wood waste and pointed out the importance of physical and chemical impurities in waste wood to improve recyclability (Faraca et al., 2019). In some other studies, waste wood has been extensively characterized for properties relevant to combustion, and the suitability of waste wood as feedstock in combustion units has also been tested (Tatàno et al., 2009) (Gehrmann et al., 2020). It was demonstrated that waste wood contained higher ash content and metals than natural virgin wood and that the chemical and physical characteristics of the different types of waste wood play a role in choosing the best use of the material as a feedstock for energy recovery. To the best of our knowledge, there are no studies examining the variability of waste wood samples using fast analytical technologies, such as Near-Infrared Spectroscopy (NIRS). In fact, Vrancken et al. have listed and reviewed different studies where sensors and modern sorting technologies were developed for recycling plants to improve/optimize the material sorting and/or measure critical waste characteristics (Vrancken et al., 2017). The optical sensors could be used to obtain real-time information about waste characteristics, which helps in selecting the best waste processes, proving to be a useful tool for stakeholders.

As it can be seen by the references cited above regarding the heterogeneity of WW, the assessment of waste wood variability is of utter importance for improving the waste management in terms of sorting and related best reuse of the material and avoiding health and environmental issues at the end of the life cycle of wood utilization. Consequently, in the current study, WW samples have been collected during a sampling in a panel board industry located in the northern part of Italy. All of the samples have been analyzed using NIRS following strict sampling protocols. Our aim is to show how the variability of WW can be characterized, both within and between each sample. Furthermore, we will show how this information can directly be implemented and used for the increased reuse of WW. Throughout the manuscript, we have decided to include information about the bound water content. This is a very important quality attribute for waste wood and is one of the most important parameters influencing the NIR analysis.

To address this issue, the following data analyses have been carried out: 1) nested analysis of variance for investigating the variability at each sampling level; 2) Principal Component Analysis (PCA) as a rapid tool for the assessment of the material variability; 3) repeated nested analysis of variance considering a subset of the original data. The first two give a good overview of the variability in and between the lots, while the latter is a good procedure for finding the most suitable sampling procedure. Obtaining information about the number of samples and replicates to be performed during sampling is fundamental to guarantee an accurate and successful application of a NIR sensor classification tool, especially when dealing with heterogeneous material. In fact, efficient quality control with a high degree of accuracy is imperative for its use in the industry. In order to meet these requirements, it is essential to have detailed information on how to perform the sampling procedure in practice, out in the field.

## Materials and Methods

### Collection of Waste Wood Samples

Waste wood samples were collected in a large panel board company located in the northern part of Italy (Lombardy region) over two days of sampling (February 18–19, 2020). The material was collected in the earliest phases of the production stream, precisely after the first step of cleaning (removal of stone, iron, and other heavy materials by washing) and grinding (reducing the particle size of the material to around 5 cm).

In order to get representative samples, a sampling plan has been defined based on the EN-15442:2011 standard (CEN, 2011). The sampling was carried out from a static lot. The material was taken every hour from the production stream in an external unloading tank for a total of 16 lots. As the incoming material is of variable quality, it is also assumed that the quality and variability within the 16 lots are different. For each lot, four representative samples were randomly taken from different locations in the lot (Mancini and Rinnan, 2021). The samples were collected using a sampling scoop for a total volume of 10 L; afterward, they were sent to the lab for the next lab and near-infrared analyses. In short, a total of 64 samples (16 lots x 4 samples from each lot) were obtained.

**FIGURE 1:**
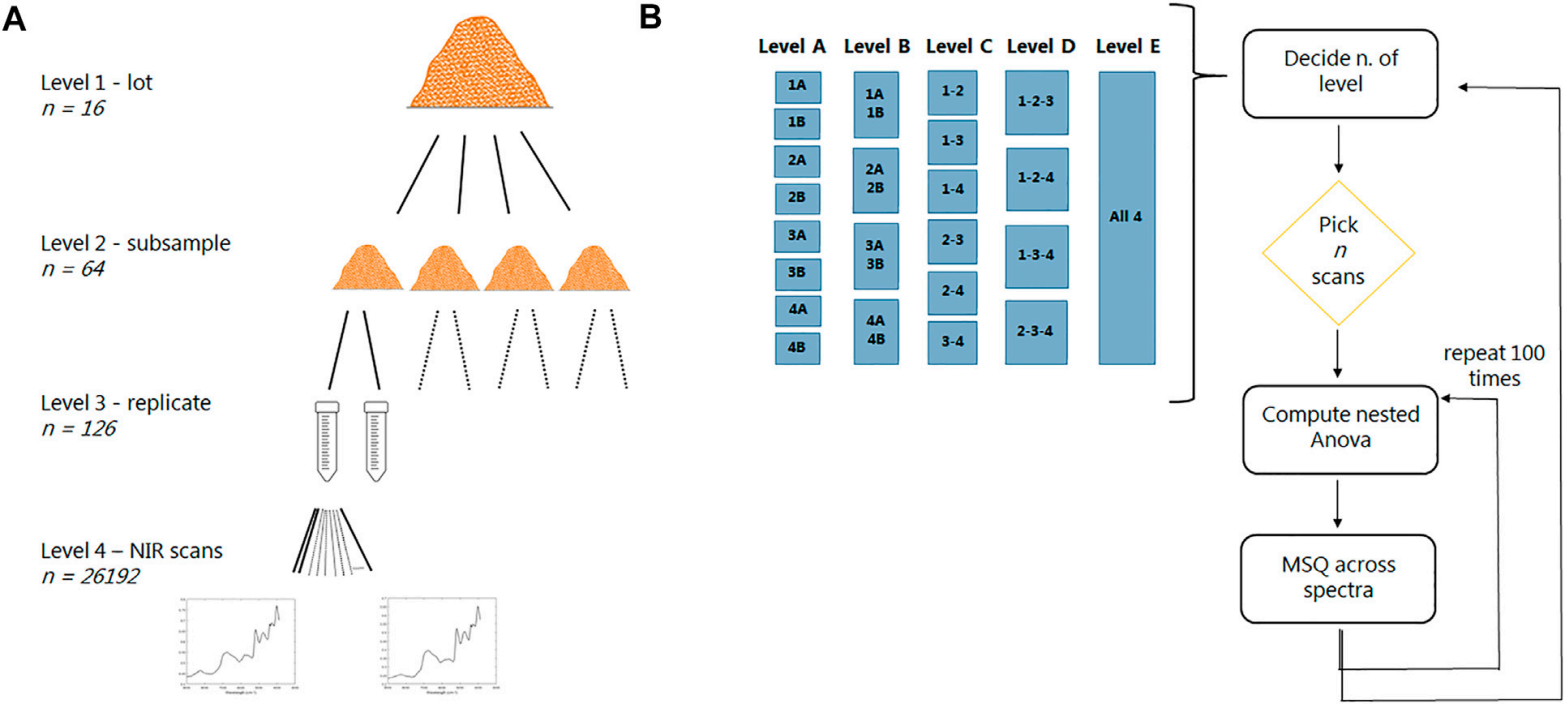
**A)** Schematic representation of the hierarchical sampling procedure. Please note that there “only” are 126 samples at level 3 due to some unfortunate problems during the NIR analysis. **(B)** Schematic representation of the data analysis procedure (*n* is the number of random scans selected). Level A, each subsample and replicate are represented equally; level B, each subsample is represented equally; level C, groups of two subsamples are represented equally; level D, groups of three subsamples are represented equally; level E scans are selected freely among all subsamples and replicates.

The hierarchical sampling procedure from lot level down the individual NIR scans is presented in Figure 1A.

### Sample Preparation

The samples have been prepared for the successive lab analysis using the technical standard UNI 15443. The sample preparation consists of a combination of sample division and particle size reduction, carefully avoiding loss in representativeness and sample composition during each step of the preparation.

Firstly, the sample has been stabilized by drying for at least 24 h not exceeding 40°C. The dried samples have been divided using a quartering process. The quartering process means that the sample is piled, divided into four, and the two opposite fractions are combined. The process of piling, dividing, and combining is repeated until the wanted sample size is achieved. Subsequently, the sample particle size has been reduced to below 5 mm using a cutting mill (mod. SM 2000; RETSCH). This material has been used for the near-infrared analysis. Finally, part of the material was further reduced to under 1 mm for the bound water content analysis. Before the NIR and lab analyses, the samples have been stored in hermetically closed plastic bags in a room with controlled temperature and humidity.

### Bound Water Content

The analytical methodology adopted for the determination of bound water content (BWC) follows the standard ISO 18122:2015. The parameter has been determined using a thermo-gravimetric analyzer (mod. 701 Leco). In detail, the sample has been air-dried to a controlled temperature (105 ± 10°C) using a muffle furnace and has been weighted until constant mass is achieved. The loss in mass has been used to calculate BWC. Each BWC value was estimated twice per sample, and the average of these estimates was used in the subsequent data analysis.

The BWC parameter has been chosen because it is easy to determine and it is important for investigating the influence of moisture in the variability of waste wood material.

### Near-Infrared Data

All waste wood samples were analyzed using a Quant FT-NIR spectrophotometer (Q-Interline A/S, Tølløse, Denmark) provided with the patented spiral sampler (Spiral Sampler, Q-Interline A/S, Tølløse, Denmark). The spiral sampler scans a total of 375 cm^2^ surface, improving the representativeness of heterogeneous samples.

The instrument is equipped with a quartz halogen lamp as a light source and an InGaAs detector. The samples were acquired in diffuse reflectance mode and were kept in rotation during the acquisition by means of the spiral sampler. Near-infrared spectra were recorded in the range from 14,885 to 3,700 cm^−1^ (equals to 670–2,700 nm) with a maximum of 210 scans per sample/tube and a spectral resolution of 8 cm^−1^. Instead of averaging all scans, each scan was stored individually, meaning that we get a good estimate of the heterogeneous nature of each sample. It is important to note that the start of each measurement had to be performed manually for each sample. Thus, some of the scans at the beginning of one series had air/plastic lids instead of the wood sample, which needed to be removed before data analysis. Random effects associated with the instrument or environment were removed by acquiring a blank spectrum, by measuring Spectralon, at the beginning of the analysis session. (However, we later realized that we should have measured this Spectralon sample several times during the measurement session, despite the whole process only taking approximately 6 h; see *Nested Analysis of Variance*.) Spectra were collected at room temperature and in duplicate for each sample in random order. The resulting dataset consists of 26,192 observations at 1,091 wavenumbers, as two tubes were only measured once due to an unfortunate computer error^1^ only realized after arriving back at the University. Consequently, level 3 of the replicate consists of 126 objects instead of 128 (see Figure 1A). The measurements were completed on the same day, taking a total of approximately 6 h.

### Nested Analysis of Variance

Considering the multi-stage approach of the sampling procedure, a nested analysis of variance (ANOVA) was computed in order to investigate the statistical differences between 1) the different lots (level 1); 2) the subsamples within each lot (level 2); 3) the two replicates within each subsample (level 3); 4) the scans within each subsample replicate (level 4).

For each sampling level, the sum of squares (SSQ) and the average of the sum of squares (MSQ) were computed (Sahai and Ageel, 2000). In detail, SSQ was computed as follows:

$$\begin{array}{l} SSQ_{lvl}\;={\sum_{n=1}^{N_{lvl}}\left(x_{n,lvl}-{\bar{x}}_{lvl-1}\right)}^{2}. \end{array}$$

Moreover, MSQ was computed as follows:

$$MSQ_{lvl}=SSQ_{lvl}/\left(N_{lvl}-N_{lvl-1}\right),$$

where *lvl* is the current level, *x*_*n,lvl*_ corresponds to the observations/average at the current level, and *N*_*lvl*_ is the number of unique measurement points at each level (e.g., number of lots for the uppermost level). The term (*N*_*lvl*_ *–N*_*lvl-1*_) thus corresponds to the degrees of freedom within each level, where *lvl-1* refers to the previous sampling level. In this way, both SSQ and MSQ are calculated to represent the individual contributions from each level of the sampling. Table 1 summarizes the computation of the degrees of freedom at each level. The MSQ was calculated for each wavenumber independently in order to investigate which wavenumbers are causing the variability at each level.

**TABLE 1:** The degree of freedom computation for the nested ANOVA. *N*_*4*_ is the total number of scans.

Levels	Degrees of freedom (D)	Computed degrees of freedom
Lot	D_1_ = N_1_−N_0_	D_1_ = 16−1
Subsample	D_2_ = N_2_−N_1_	D_2_ = 64−16
Replicate	D_3_ = N_3_−N_2_	D_3_ = 126−64
Scan	D_4_ = N_4_−N_3_	D_4_ = 26,192−126
Total	D_Tot_ = N_4_−N_0_	D_Tot_ = 26,192−1

Before any variance analysis, the NIR spectra have been preprocessed by Multiplicative Scatter Correction (MSC) (Martens et al., 1983) in order to reduce the light scattering effects (Rinnan et al., 2009).

### Deciding the Best Sampling Procedure

In order to find the best sampling procedure to describe the variability of waste wood material, the nested analysis of variance was computed again considering the setup reported in Table 2. Based on the total number of scans for each of the tested levels, the nested analysis of variance was computed again, taking *n* random selected scans, and the procedure was repeated one hundred times for each of the new levels.

**TABLE 2:** Setup for the computation of the nested analysis of variance for deciding the best sampling procedure.

	Setup	Total n. of scans	n. of randomly selected scans (*n*)
Level A	A single subsample with replicates as two different subsamples	210	25, 50, 75, 100, 125, 150
Level B	A single subsamples with replicates together	420	25, 50, 75, 100, 150, 200, 250, 300
Level C	Two subsamples	840	25, 50, 100, 150, 200, 300, 400, 600
Level D	Three subsamples	1,260	25, 50, 100, 150, 250, 400, 600, 900
Level E	All 4 subsamples	1,680	25, 50, 100, 150, 250, 500, 750, 1,000

This is important, as how to perform the sampling procedure in the real world is of utter importance for the usefulness of applying advanced sensors to the system of WW reuse. Here, we investigated how the variability of the lot is described by increasing the number of subsamples and/or scans. We have decided to perform this at different levels of constraints, efficiently showing the effect of each of these constraints on the subsequent sampling conclusion. In detail, at level A, each subsample and replicate are represented with the same number of scans; at level B, each subsample is represented with the same number of scans; at level C, two subsamples are grouped together; at level D, three subsamples are grouped, while level E picks scans at random across all subsamples and replicates. Differences and similarities between these different approaches will aid in finding the optimal sampling procedure, with regard to both the number of subsamples and replicates and number of scans necessary to cover the variability. A schematic representation of the data analysis procedure is displayed in Figure 1B.

### Multivariate Data Analysis

Principal Component Analysis (PCA) (Wold et al., 1987) has been computed using two different datasets: the mean-centered MSQ values of the nested analysis of variance and the preprocessed NIR absorbance values of the waste wood samples.

The former was performed in order to investigate similarities in the variability among the lots at the different sampling levels. We are well aware that this is an untraditional use of PCA, but it gives a nice and quick overview of how the variability varies between the lots. The latter was performed in order to explore the variability of waste wood and search for differences/groupings among the lots at each sampling level. In this latter case, the computation was carried out on the MSC pretreated and mean-centered data. In order to search for differences among the lots and investigate the variability within each lot, a confidence ellipse is drawn around each lot. This ellipse is calculated based on a local PCA on the scores, indicating the direction and extent of variability for each lot individually. Each ellipse was calculated using the mean score values as the center, and the standard error of each variability direction as the radius of the ellipse. The loading plot of the two first PCs was investigated to identify the compounds associated with the variability of the waste wood samples and the variability within the lots.

Both the multivariate data analysis and the nested analysis of variance have been computed using Matlab software (ver. MATLAB R2019b, The MathWorks) with in-house functions based on existing algorithms.

## Results and Discussion

### Spectra

**FIGURE 2:**
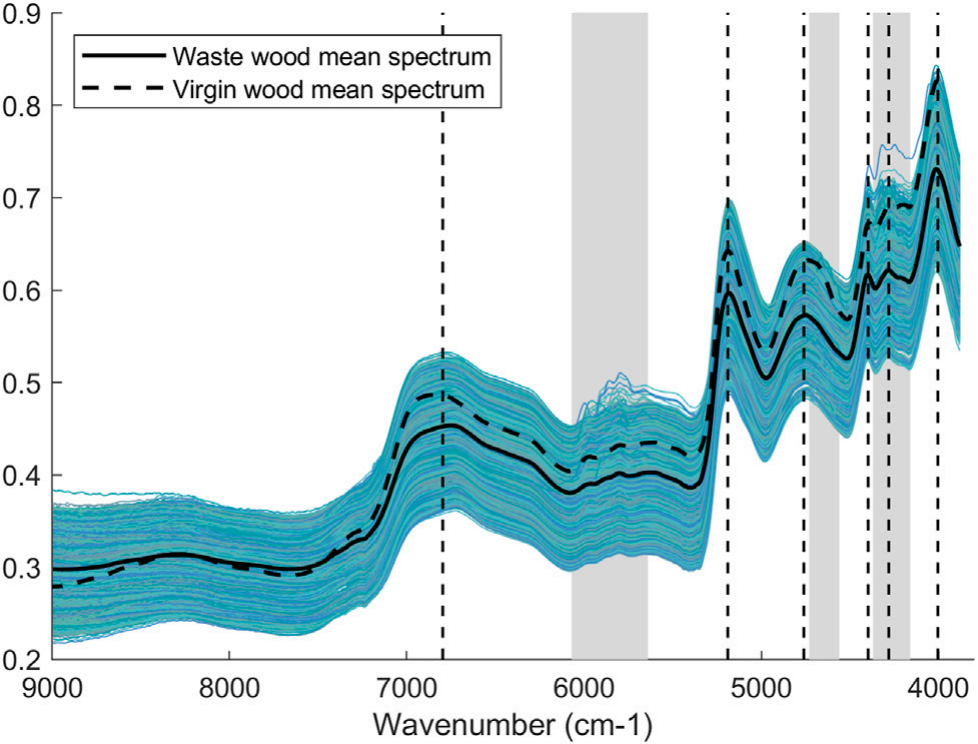
All the spectra of waste wood samples with the mean spectrum of all the waste wood samples highlighted with a solid black line, and the mean spectrum of virgin wood samples highlighted with a dotted black line. Vertical dotted lines refer to the most relevant wavenumbers and are also reported in Table 3. The grey areas highlight the spectral areas mostly associated with glue compounds.

A total of 55 spectra was detected as either being due to the plastic lid or air, and was deleted before any further data analysis. Furthermore, wavenumbers lower than 3,880 cm^−1^ and greater than 9,000 cm^−1^ were removed as the data were either deemed noisy or containing very limited information. The new dataset thus consists of 26,192 scans measured at 664 wavenumbers. Figure 2 illustrates the plot of all the spectra of waste wood samples and their mean spectrum highlighted with a solid black line. Because of the light scattering, all the spectra have been preprocessed with MSC before any further data analysis. In addition, in order to investigate the differences between waste wood and virgin wood, the mean spectrum of virgin wood samples was added to Figure 2 as a dotted black line. The virgin wood samples have been acquired during a previous study (Toscano et al., 2017). The most relevant wavenumbers in the two spectra are marked with vertical dotted lines and reported in Table 3. As it can be noted, the same spectral wavenumbers selected for the mean spectrum of waste wood samples can also be found in the mean spectrum of virgin wood samples, demonstrating similar chemical composition. By inspecting the waste wood spectra, we can clearly see that some spectral areas include observations with deviating trends: 6,070–5,640 cm^−1^, 4,730–4,560 cm^−1^, and 4,370–4,160 cm^−1^, strongly indicating that it will be possible later to classify the samples between virgin wood and treated wood. These spectral areas are probably associated with glue compounds related to the composite wood materials or plastic materials contained in the waste wood.

**TABLE 3:** Near-infrared absorption band assignment associated with the most important wavenumbers (str.: stretching; def.: deformation; OT: overtone; L: lignin; H: hemicellulose; C: cellulose).

Measured wavenumber (cm^−1^)	Bibliography wavenumber (cm^−1^)	Compound	Assignment
6,797	6,790	C	1st OT O-H str. Schwanninger et al. (2011)
	6,800	H	1st OT O-H str. Schwanninger et al. (2011)
5,189	5,220–5,150	Water	O-H asymmetric str. + O-H def. Of H_2_O Schwanninger et al. (2011)
4,760	4,762	C	O-H and C-H def. + O-H str. Sandak et al. (2010)
	4,780–4,760	C	O-H and C-H def. + O-H str. Schwanninger et al. (2011)
	4,890–4,620	C	O-H str. + C-H def. Schwanninger et al. (2011)
4,397	4,392	C	O-H str. + C-C str. and/or C-H str. + C-H def. Schwanninger et al. (2011)
4,281	4,288	H	C-H str. + C-H def. Schwanninger et al. (2011)
	4,280	C	C-H str. + C-H def. Schwanninger et al. (2011)
	4,280	L	C-H str. + C-H_2_ def. Schwanninger et al. (2011)
	4,282	C	C-H str. + C-H_2_ def. combination band (and 2nd OT of C-H_2_ str.) Hein et al. (2011)
4,004	4,014	L	C-H str. + C-C str. Schwanninger et al. (2011)

As reported by Lian et al., the band at 5,911 cm^−1^ corresponds to the characteristic absorption peak of C-H in methyl glycol, while the peak at 5,996 cm^−1^ corresponds to C-H on the benzene ring (Lian et al., 2020). In general, the spectral range between 6,700 and 6,330 cm^−1^ corresponds to the characteristic absorption of methyl glycol, indicating that it is related to glue/plastic compounds. Furthermore, these results were confirmed in a study by Workman and Weyer, where the assigned peaks at 5,847 and 5,975 cm^−1^ are attributed to C-H from methyl of glue, while the band at 5,624 cm^−1^ was assigned as the second overtone of CH methylene of glue (Workman and Weyer, 2007). The band at 5,805 cm^−1^ was assigned to the 1st overtone of C–H stretching of methyl and methylene structures of glue (Tomlinson et al., 2006). Regarding the second spectral area, the absorption band at 4,440 cm^−1^ is related to the CH_2_ combination of methylol group (Dessipri et al., 2003). In another study, Hein et al. have investigated the physical and mechanical properties of agro-based particleboards by NIR spectroscopy and assigned the peak at 4,587 cm^−1^ to symmetric NH stretching and NH_2_ rocking and/or 2nd overtone of amide I and amide III (Hein et al., 2011). Moreover, the relationship between this spectral region and wood composite materials is confirmed by the peak at 4,617 cm^−1^, associated with NH_2_ species from urea (Dessipri et al., 2003), and 4,550 cm^−1^ assigned to NH symmetrical stretching and NH bending combination bands (Henriques et al., 2012). Lastly, the region from 4,370 to 4,160 cm^−1^ is assigned to the combination band of NH_2_ and CH bonds.

The knowledge of the chemical composition of the waste wood and the inspection of the spectra are important steps for defining the waste wood quality and, accordingly, the best reuse of the material. The difference between the mean spectra of virgin wood and waste wood indicates that some absorption bands of the two materials are not exactly the same, suggesting that a classification model for separating the material according to its best reuse would perform well.

### Bound Water Content Analysis

A descriptive statistic of the BWC has been carried out. The 64 waste wood samples analyzed have a mean = 8.0%, standard deviation = 0.7%, max value = 11.1%, and min value = 7.0%. Thus, the parameter has a range of 4.1%. An outlier sample in BWC values has been detected using Tukey’s test. The test identifies the possible outliers of the samples falling outside the Q1 - 1.5 · IQR (interquartile range) or the Q3 + 1.5 · IQR limits; Q1 and Q3 are first and third quartiles, respectively. For this study, limits that are more conservative have been used: Q1 - 3.0 · IQR or Q3 + 3.0 · IQR. The lot with the highest variability in BWC was lot 12 (range of 2.92%), and the one with the lowest was lot 15 (range of 0.24%). The average lot variability in BWC was 0.79%. The reported results are useful for the discussion of the successive outcomes (see *Nested Analysis of Variance* and *PCA*).

A nested analysis of variance was also computed. The MSQ value is higher at lot level (MSQ = 3.20), decreases considerably at subsample level (MSQ = 0.39), and drops even further at the replicate level (MSQ = 7.11 e-4). The results confirm that by increasing the number of samples, the variability in their moisture content also decreases.

### Nested Analysis of Variance

**FIGURE 3:**
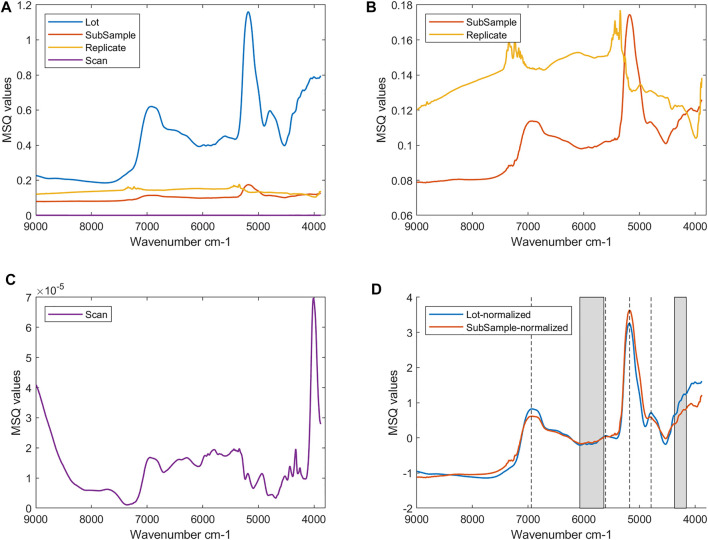
The MSQ values of the nested ANOVA: **(A)** all sampling levels, **(B)** only subsample and replicate levels, **(C)** only scan level, and **(D)** the normalized MSQ values at the lot and subsample sampling levels (the grey areas highlight the spectral areas mostly associated with glue compounds).

The nested ANOVA was computed on the dataset consisting of 26,192 observations and 664 wavenumbers. The analysis of variance has been computed on the spectra preprocessed with MSC on all the sampling levels. Figure 3 (A, B, and C) shows the plot of the MSQ values plotted against the wavenumbers at the different sampling levels. As expected, the variability is higher at the lot level (Figure 3A) and lowest at the scan level (Figure 3C). Unexpectedly, the variability at the subsample level is lower than at the replicate level (Figure 3B) and will therefore be investigated further. The subsample and lot lines have a similar trend indicating that the variability is affected by the same wavenumbers. To better investigate this, Figure 3D shows the normalized MSQ values at the lot and subsample levels. The two lines differ for some wavenumbers. In detail, the lot level has two higher and sharper peaks at 5,609 cm^−1^ and 4,791 cm^−1^. The former is assigned to 1st overtone of CH_2_ stretching of cellulose, while the latter is related to OH stretching + OH and CH deformation of cellulose and hemicellulose (Schwanninger et al., 2011). Both lines have a high absorption band at 5,177 cm^−1^ (O-H stretching and O-H deformation of H_2_O) and 6,943 cm^−1^ (first overtone O-H stretching of water), indicating that the bound water content plays a role in the variability of the waste wood material, as also confirmed by the results reported in *Bound Water Content*. The subsample level has two noisy areas: between 7,400 and 7,050 cm^−1^ and between 5,500 and 5,200 cm^−1^. Finally, we can observe small “vibrations” in the areas 6,070–5,640 cm^−1^ and 4,370–4,160 cm^−1^, confirming our previous conclusions (see Figure 2).

**FIGURE 4:**
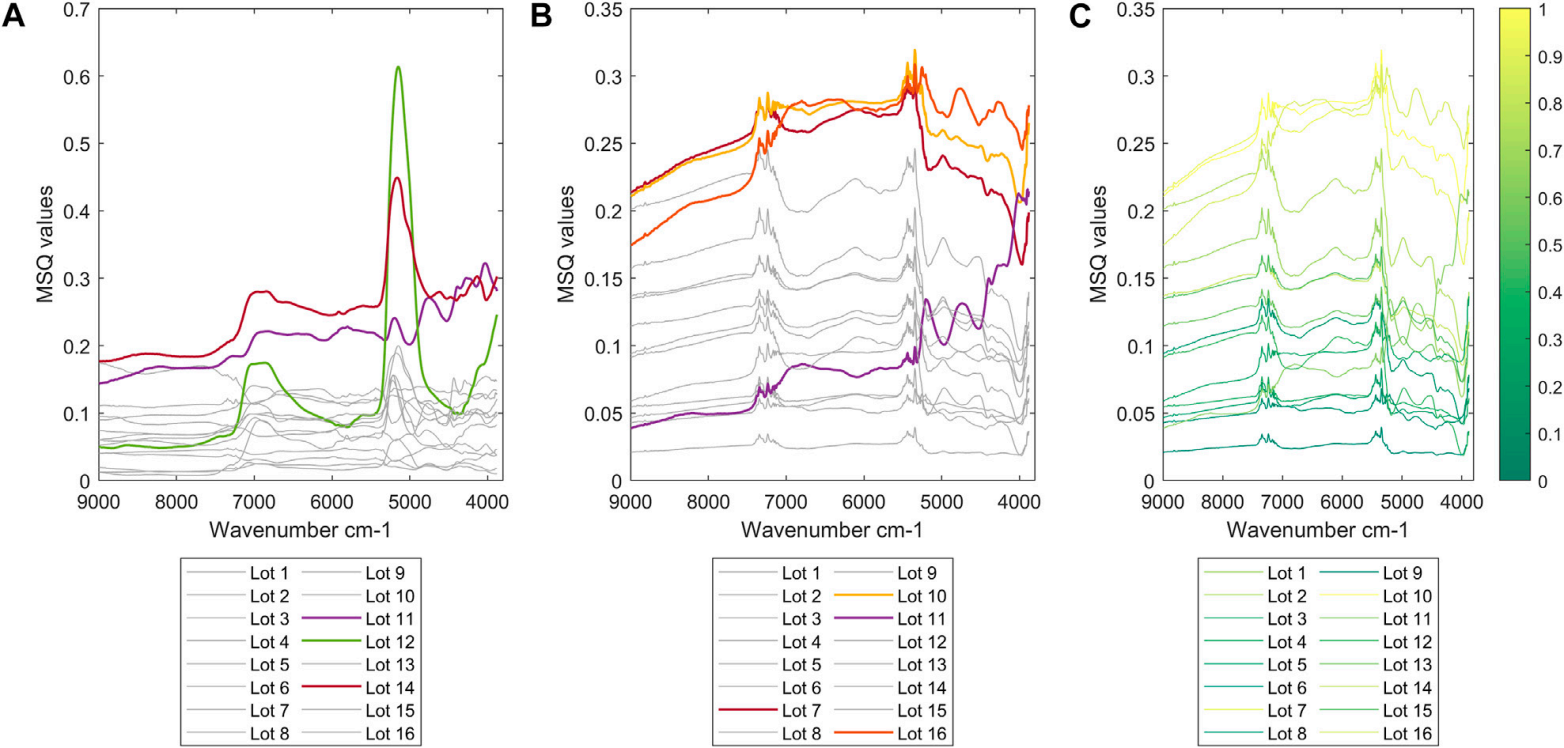
The MSQ values of the nested analysis of variance computed within each lot **(A)** at subsample level and **(B)** replicate sampling level. MSQ values at the replicate sampling level are also colored according to the distance in the PCA score plot between the two replicates of a sample **(C)**.

Figure 4 shows the plot of the MSQ values for each of the 16 lots at the subsample and replicate levels, respectively. Basically, the nested ANOVA has been computed again for each of the 16 lots individually, and the MSQ values have been estimated at both the subsample and replicate levels of sampling. This gives an indication about the variability among the different lots. In Figure 4A, it can be noted that the lots with higher variability are lots 12, 14, and 11. In detail, lot 12 has a higher variability at wavenumber 5146 cm^−1^, while MSQ values of lots 14 and 11 are higher on all the other wavenumbers. The band at 5,146 cm^−1^ is assigned to O-H asymmetric stretching and O-H deformation of H_2_O (Schwanninger et al., 2011), indicating that the higher variability of the lot is probably related to a higher BWC in some samples. In fact, lot 12 contains the sample with the highest BWC value (11.1%) (see *Bound Water Content*).

Figure 4B reports the variability between the two replicates of the subsamples within each lot. Lots 16, 10, and 7 (in descending order) have higher MSQ values. The MSQ values of lot 11 are quite different, resulting in a particular shape/trend of the variance line, more similar to a spectrum. All the other lots show higher variability in the wavenumbers between 7,400 and 7,050 cm^−1^ and between 5,500 and 5,200 cm^−1^. The two spectral regions are quite noisy and the peaks do not probably contain relevant information. However, they could be related to the detector drift since, unfortunately, only one reference spectrum at the very beginning of the analysis was acquired (see *Bound Water Content*). The differences in the variability among the lots could be explained by calculating the distance in the PCA score plot (see *PCA* section) between the two replicates at the subsample level. Figure 4C shows the lots colored according to the replicates distance and we can conclude that the longer the distance between the two replicates in the PCA score plot, the higher the MSQ values and, consequently, the variability at the replicate level.

### PCA

**FIGURE 5:**
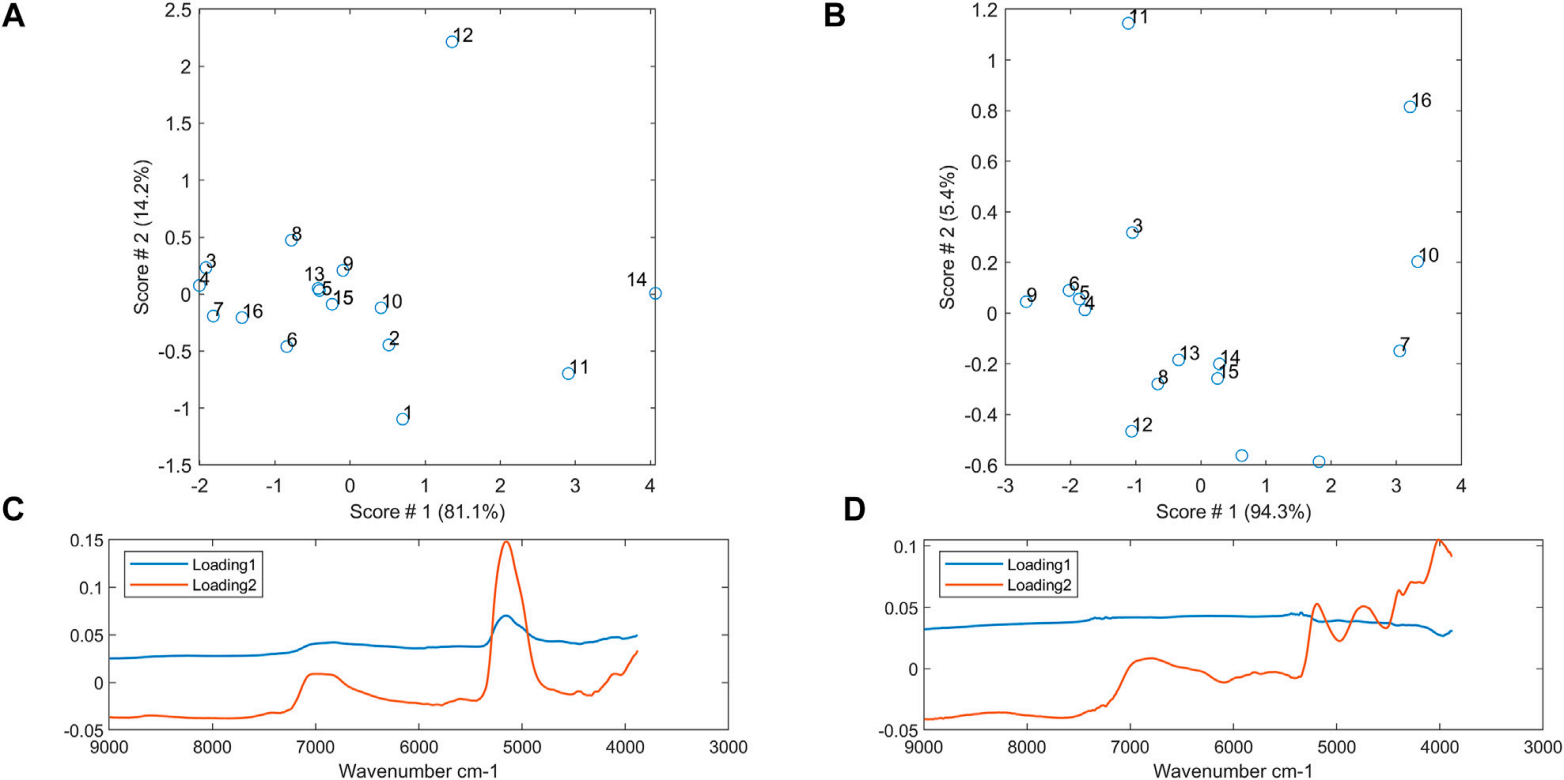
PCA score plot of MSQ values of the nested analysis of variance at **(A)** subsample level and **(B)** replicate level. PCA loading plot of MSQ values of the analysis of variance **(C)** at subsample level and **(D)** replicate level.

In order to get a quick overview of how the variability changes between the different lots, a PCA was carried out using the MSQ values of the nested analysis of variance, computed individually for each lot, at both subsample and replicate levels of sampling. The score plot confirms the results of the nested ANOVA, but with increased clarity. At the subsample level (Figure 5A), the lots with the most deviating scores are 11, 12, and 14, while at the replicate level (Figure 5B), lots 7, 10, 11, and 16 deviate the most compared to the remaining lots.

The loadings were investigated to understand what variables are responsible for the separation of the lots in the PCA scores plots. At the subsample level (Figure 5C), both the first and second PCA loadings show two main bands at around 6,950 cm^−1^ and 5,150 cm^−1^. Both bands are related to the overtone of O-H stretching bonds (Schwanninger et al., 2011), confirming the results of the nested analysis of variance and what already was stated during the discussion of Figure 4. At the replicate level (Figure 5D), the first loading shows the same noisy areas (i.e., between 7,400 and 7,050 cm^−1^ and between 5,500 and 5,200 cm^−1^), as shown in Figure 4. The second loading contains information related to the variability of lot 11. It is important to note that the PCA analysis on MSQ values confirmed the outcomes of the nested analysis of variance and is an efficient alternative, giving a nice and quick overview of how the variability varies among the different lots.

**FIGURE 6:**
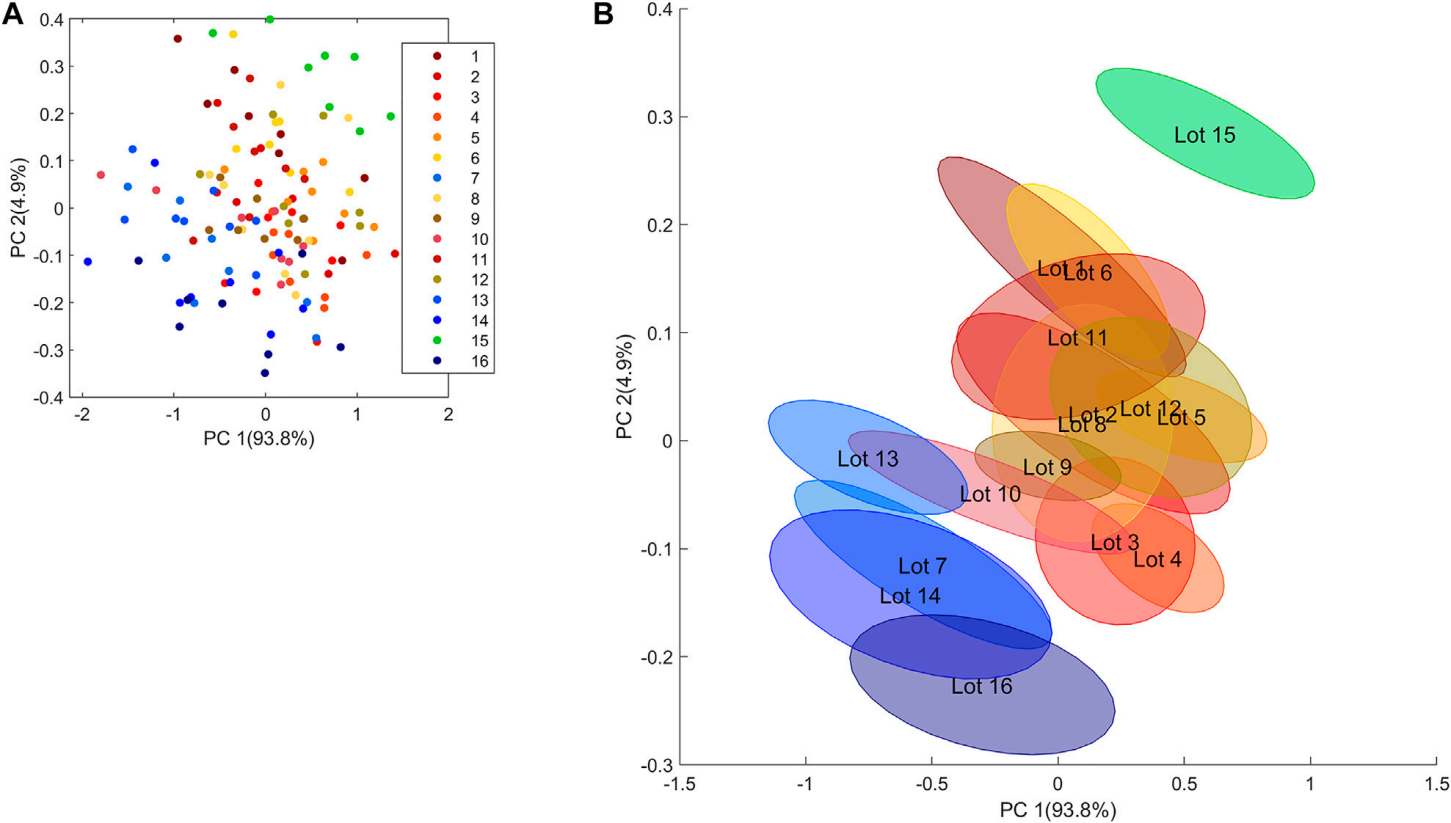
PCA on the average spectra at the subsample level. Each lot has been colored in shades of blue, red, and green. The color scheme is based on the position of the lots in the score space. **(A)** The first two scores colored according to the lot number and **(B)** PCA score plot of the waste wood sample with standard error ellipses for each lot.

To explain why some replicates/subsamples present a higher variability than others, the average spectra at the subsample level, after preprocessing with MSC, have been taken into account. A PCA was computed based on these 16 x 8 spectra, and as it can be noted in the PCA score plot (Figure 6A), the samples seem to be spread across the whole score space without any clear groupings between them. However, by closer inspection, there is some trend in the distribution of the samples according to the lot; i.e., the samples with higher BWC are located in the bottom right part of the score plot (i.e., lots 4 and 5 and some samples of lot 12).

In order to get a clearer picture of the differences among the lots, confidence ellipses were computed using the standard error for each lot. The score plot of the two first PCs clearly shows some groupings among the lots (Figure 6B, please note that this is the same plot as Figure 6A, but now with confidence ellipses instead of each individual subsample being plotted). Lot 15 is clearly different from the others (lower range in BWC). Lots 7, 13, 14, and 16 are located at the bottom left part of the PCA score plot. All the other lots are close to each other and located in the central part of the score plot, indicating that their composition/variability is very similar. The size of the ellipses confirms that the lots with the highest variability are lots 8, 11, and 12, and 14. Lots 4 and 9 have the lowest variability, confirming once again the results of the nested ANOVA.

### Deciding the Best Sampling Procedure

**FIGURE 7:**
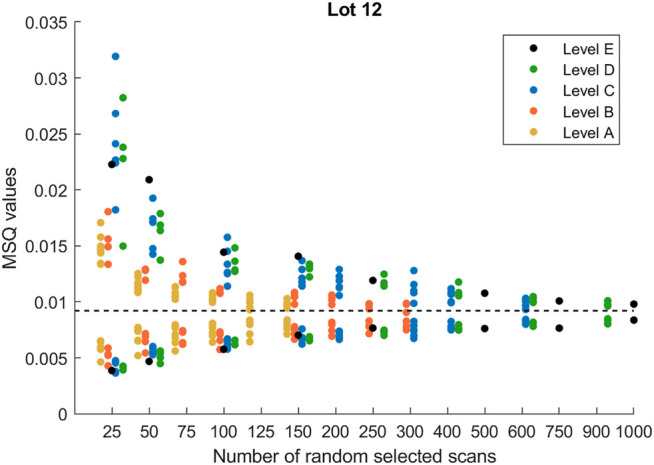
Variability in the MSQ values of nested analysis of variance changing the number of randomly selected scans and the number of subsamples. The dotted horizontal black line represents the estimated overall variability of lot 12. Level A, each subsample and replicate are represented equally; level B, each subsample is represented equally; level C, groups of two subsamples are represented equally; level D, groups of three subsamples are represented equally; level E scans are selected freely among all subsamples and replicates. For a detailed description of the different levels, please refer to Figure 1B.

For the practical implementation of a NIR sensor classification tool in the WW industry, it is imperative to know how to actually perform the NIR measurements in order to ensure representative and reliable measurements of the heterogeneous WW material. In this section, we will give strong indications in this regard by describing the variability of waste wood material with a nested ANOVA with resampling. The analysis was performed on all the 16 lots and all showed similar results. However, in order to simplify the discussions, we will focus our analysis on one lot only. We have decided to report lot 12 as an example because our earlier results indicated that this is the one with the highest variability. Figure 7 shows the variability in the MSQ values of nested ANOVA at each of the aforementioned levels (see *Deciding the Best Sampling Procedure*). As noted, the variation decreases with increasing number of scans, as expected. This trend is the same in all five levels.

These results provide good indications regarding the optimal sampling procedure to carry out in terms of the number of subsamples and scans to be performed to describe the variability in the waste wood materials. In fact, the variability in the MSQ values reached almost constant values at 125 scans for level A; 250 scans for level B; 400 scans for level C; 600 scans for level D; around 500 for level E. It means that the same variability can be obtained by increasing the number of subsamples and decreasing the number of scans or by decreasing the number of subsamples and increasing the number of scans.

As seen from Figure 7, levels A and B give lower variability than the remaining sampling procedures, clearly indicating that it is insufficient to investigate one subsample only. This is confirmed by the MSQ values located slightly below the horizontal black line, which is the estimated overall variability of lot 12 and is deemed to be the true estimated variability of the lot. The three other sampling schemes are all very similar, giving indications that taking out two subsamples, splitting them into two replicates, and then measuring each of them with at least 100 scans seem to provide reliable and representative variability estimates of the lots (around 10 m^3^ of fairly heterogeneous waste wood material).

## Conclusion

Waste wood samples were collected in a panel board industry located in the northern part of Italy. All samples were analyzed using FT-NIR provided with a spiral sampler to investigate their variability and heterogeneity. A nested analysis of variance was computed to investigate the statistical differences for each level of the sampling procedure, i.e., lot, subsample, replicate, and scan levels. According to the results, waste wood has the highest variability at the lot level and lowest at the scan level.

PCA analysis on the MSQ values of the nested analysis of variance confirms the results of the nested ANOVA with increased clarity and shows how some lots deviate more from the others. The score plot clearly shows groupings among the lots and the loading plot displays that the main bands responsible for such separation are related to the overtone of O-H stretching bonds, which we also were able to confirm through reference analysis.

The knowledge of waste wood variability and composition is a key point for enhancing the sorting and related best reuse of the material with related positive effects in terms of economic, health, and environmental issues. NIRS proves to be a useful technique for rapidly obtaining this information. The definition of the most appropriate sampling procedure is essential for improving waste wood management and moving NIRS into real industrial applications. In fact, having a number of samples, replicates, and scans able to describe the variability of the material translates into reliable analytical results and accurate classification models for sorting the material based on the best reuse, especially when dealing with heterogeneous material. This study has proved that by taking at least two subsamples, splitting them into two replicates, and measuring each of them with at least 100 NIR scans, it is possible to describe the variability of around 10 m^3^ of waste wood material. In future studies, this result can be used as the starting point for developing classification models, essential for more accurate and sustainable waste wood management.

These results have a large potential impact on the waste management sector, representing the first steps for moving NIR sensors to industrial waste management applications. In fact, the methodology used in this study can be applied not only to any other NIR spectrophotometers but also to other waste sources. When working with waste in general, the big challenge is the heterogeneity of the material. Thus, having a protocol that ensures efficient and reliable sampling will lead to the success of the subsequent classification of the waste according to waste categories, which will improve the sorting and, as a consequence, the reuse of the material.

## Data Availability

The datasets presented in this study can be found in an online repository. The name of the repository and accession number can be found as follows: the dataset is uploaded to Zenodo repository with data DOI’s assignment (http://doi.org/10.5281/zenodo.4896579).

## Footnotes

1 For two sample replicates, the computer froze without saving the collected data.
